# Subsequent Cancers following Non-myeloablative Conditioning for Allogeneic Hematopoietic Cell Transplantation

**DOI:** 10.21203/rs.3.rs-5760731/v1

**Published:** 2025-01-08

**Authors:** Phuong Vo, Kevin Ng, H. Gary Schoch, Jason Cooper, Abhishek Vupalanchi, Mary Flowers, Brenda Sandmaier, Ted Gooley, Rainer Storb

**Affiliations:** Fred Hutchinson Cancer Center; Fred Hutchinson Cancer Center; Fred Hutchinson Cancer Research Center; University of Washington; University of Washington; Fred Hutchinson Cancer Center; Fred Hutchinson Cancer Center; University of Washington, Seattle, Washington, United States of America; Fred Hutchinson Cancer Research Institute

## Abstract

We examined the risk of subsequent malignant neoplasms (SMNs) in 1720 patients with hematologic cancers given allogeneic hematopoietic grafts from 03/1998 to 08/2023 after nonmyeloablative conditioning regimens. With a median follow-up of 12 years, the cumulative incidence of SMNs was 17% (95% CI, [15%, 19%]). Most SMNs (n = 543) were non-melanoma skin cancers seen in 208 patients; unfortunately, information on these cancers was not available in the Surveillance, Epidemiology, and End Results (SEER) database for comparison with such tumors in the general population. However, developing non-melanoma skin cancers was statistically significantly associated with chronic GVHD and, thus, unlikely to be conditioning regimen related. Eighty-six patients (5%) developed 93 other SMNs. This number (93 SNMs) significantly exceeded the expected 73.4 cases in the comparison group (p = 0.03). This increase was driven exclusively by increases in uterine adenocarcinoma (n = 2), squamous lip cancer (n = 5), and squamous penile cancer (n = 2); the latter two cancers were, again, associated with chronic GVHD. Apart from these three tumor types, there were no observed increases in the risk of other tumors compared to those in the general population.

## INTRODUCTION

Preclinical studies led to the development of a minimal intensity regimen to condition older or medically unfit patients with hematological malignancies for allogenic hematopoietic cell transplantation (HCT) [1, 2]. Conditioning consists of fludarabine combined with 2 or 3 Gray (Gy) total body irradiation, though in occasional patients fludarabine was complemented or replaced by other drugs. A then novel post grafting immunosuppressive drug combination consisting of mycophenolate mofetil (MMF) combined with a calcineurin inhibitor served both to enable sustained engraftment and control GVHD. While the regimen is minimally intense, concerns existed that it might increase the risk of subsequent cancers in these mostly elderly patients. To address these concerns, we carried out a retrospective analysis of such cancers among 1720 patients with a follow-up for 0.1–25 (median 12) years after allogenic HCT. The cancer incidence among this patient cohort was compared to that among an age-matched U.S. population captured in the Surveillance, Epidemiology, and End Results (SEER) database [3].

## METHODS

### Patients

Records of 1720 patients with hematologic malignancies who underwent non-myeloablative allogeneic HCT at the Fred Hutchinson Cancer Center (Seattle, WA) between 03/1998 to 08/2023 were reviewed. 1465 (85.2%) patients were transplanted on prospective trials which, since its inception, have been registered on ClinicalTrials.Gov. 255 (14.8%) patients were transplanted under a standard treatment plan. Patients who underwent transplantation prior to the year 2014 were incorporated into a preceding analysis conducted by Baker et al. [11]; however, they were aggregated with other patients given higher total body irradiation (TBI) doses; moreover, that study was focused on comparing tumor incidences among various conditioning regimens. All current patients signed consent forms including permission for long-term follow-up. All underwent conditioning with non-myeloablative regimens, consisting of 2–3 Gy (with 1303 patients receiving 2 Gy and 417 patients receiving 3 Gy), either alone or in combination with immunosuppressive chemotherapy such as fludarabine, low-dose cyclophosphamide/fludarabine, or clofarabine, as detailed in Table 1. Graft-versus-host prophylaxis is also indicated in Table 1. The median age of patients at the time of transplant was 59.9 years with range of 2.6 to 80.9 years.

### Patient Follow-Up and Data Collection

Patients are followed for life in the Long-term Follow-up Program under a standardized protocol approved by the Institutional Review Board. In addition to patient characteristics, details of HCT conditioning regimens and the early post-HCT course, collected prior to discharge from the active service, information on late events, including the development of secondary malignancies are prospectively collected and maintained in the database. Patients are invited to return to Seattle for a comprehensive medical evaluation at one-year after HCT and again thereafter, if clinically indicated. In addition, on an annual basis, health status questionnaires are sent both to patients and referring physicians to obtain additional details on late effects, including secondary cancers which are verified by physicians’ reports and, whenever possible, by pathology as well as surgical and other reports for confirmation.

### Statistical methods

The probability of SMN was summarized using cumulative incidence estimates, where death without a SMN was regarded as a competing risk. Standardized incidence ratios (SIRs) were calculated as ratios of observed numbers of SMNs to expected in the SEER program (SEER Cancer Statistics Review [CSR], 1998–2023) [4]. P-values were estimated by inverting the confidence intervals proposed by Sahai and Khurshid [5, 6] as mentioned in the SEER Stat Help System [7]. Given that data on basal/squamous cell skin cancers are not recorded in the SEER base, all statistical calculations, presented under Results, pertain to other cancers. Figures were generated using the ggplot2 package in R Version 4.3.2 [8].

## RESULTS

### Patient characteristics

Patient and donor characteristics are shown in Table 1. One hundred and nine patients had the diagnosis of Hodgkin disease (6.3%); 308 (17.9%) of non-Hodgkin lymphoma; 570 (33.1%) of acute myeloid leukemia (AML); 270 (15.7%) of myelodysplastic syndromes (MDS); 214 (12.4%) of multiple myeloma (MM); 112 (6.5%) of acute lymphoblastic leukemia (ALL) and 137 (8.0%) of chronic lymphocytic leukemia (CLL). Among the patients, 39.6% underwent HLA-matched related donor transplants, while 60.4% received grafts from unrelated volunteer donors (38.9% HLA matched and 21.5% HLA-mismatched). The prophylaxis for graft-versus-host disease (GVHD) used a calcineurin inhibitor (CNI) in combination with mycophenolate mofetil (MMF) alone (76.9%), or with added sirolimus (15.3%) or post-transplant cyclophosphamide (7.8%).

### Subsequent cancers

#### All cancers

**Figure 1:**
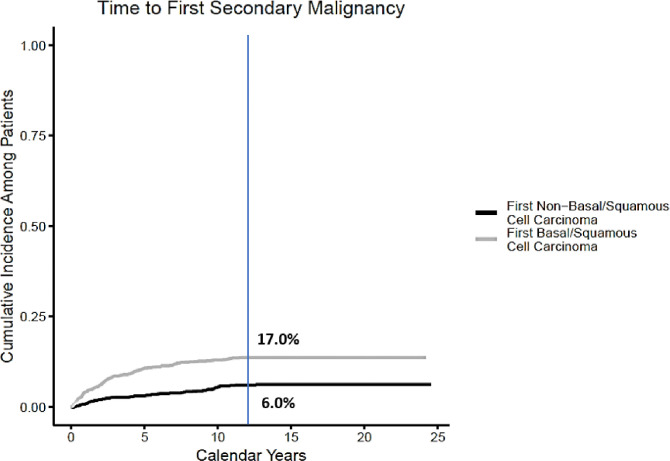
The cumulative incidence of all observed SMNs.

With a median follow-up of 12 (range 0.1–24) years, a total of 636 cancers were seen in 256 patients. Of the cancers, 543 were basal/squamous cell skin cancers diagnosed in 208 patients and 93 were other cancers diagnosed in 86 patients (Table 2). The cumulative incidence of basal/squamous cell carcinomas, based on time to initial SMN, at 12 years was 17% (95% CI, [15%, 19%]) (Fig. 1; black curve. The cumulative incidence of SMNs excluding basal/squamous cell skin cancers was 6% (95% CI, 4.9%−7.4%) at 12 years (Fig. 1; grey curve).

#### Basal and squamous cell skin cancer

Two hundred and eight patients developed a total of 543 basal/squamous cell skin cancers. These cancers were predominantly seen among patients with chronic GVHD; among 754 patients with chronic GVHD, 110 patients developed at least 1 basal/squamous cell skin carcinoma, 608 of those patients never developed basal/squamous cell skin carcinoma, and 36 developed basal/squamous cell skin carcinoma prior to their chronic GVHD. Among the 966 patients without chronic GVHD, 62 developed at least 1 basal/squamous cell skin carcinoma while 904 never developed basal/squamous cell skin carcinoma.

A fitted univariate cause-specific Cox proportional hazards model showed statistically significant evidence that the hazard rate of basal/squamous cell carcinoma among patients with chronic GVHD was greater than that among those without chronic GVHD (hazard rate 1.5 [95% CI: 1.07, 1.99]; p < 0.05).

### Other cancers

Eighty-six patients (5%) developed 93 other SMNs under SEER Recodes, with a cumulative incidence at 12 years of 6.0% (95% CI, [4.9%, 7.4%]). The nature of these SMNs is shown in Table 2. There were 7 patients who developed more than 1 SMNs, excluding basal cell and squamous cell skin cancers (Table 3).

The incidence of these SMNs (93 cancers) was statistically significantly greater than the expected incidence of 73.4 (p = 0.03). That difference was driven exclusively by higher-than-expected incidences of uterine cancer (n = 2; 87.7 times greater; (95% CI: 10.62–316.87, p < 0.01), lip cancer (n = 5; 25.3 times greater; (95% CI: 8.21, 59.01, p < 0.001) ), and penile squamous cell cancer (n = 2; 15.1 times greater; (95% CI: 1.82–54.37, p = 0.016)). Of note, 5 out of 7 patients with lip and penile cancers had a history of chronic GVHD. As for the 2 patients with uterus cancer, their tumors were diagnosed 2 and 9 years post-HCT, respectively. One of the two patients was 70-year-old and had a history of breast cancer and thyroid cancer before HCT; she also had recurrence of thyroid cancer after HCT and died a year later. The second patient was also 70 years old and has remained in remission 5 years after surgical removal of the tumor.

There were no statistically significant increases in the risk of any of the other malignancies including leukemia and MDS (classified under miscellaneous) (Table 2). One of 2 cases of AML was a relapse in recipient cells and 1 had AML in donor cells. Six patients developed MDS; 4 of these were relapses in recipient cells and two new malignancies in donor cells.

## DISCUSSION

The introduction of nonmyeloablative conditioning regimens in 1998 has expanded the use of allogeneic HCT considerably to include older and medically unfit patients with hematologic malignancies. This transplant approach relied nearly entirely on graft-versus-tumor effects for eradication of the underlying malignancies. Thorough follow-up of 1720 patients for up to 24 years after allogeneic HCT enabled the current study to assess the transplanted patients’ risk of SMNs and compare that risk to the risk of cancers in the general population.

The predominant tumors seen among our patients were squamous cell and basal cell skin cancers with 208 patients developing 543 such cancers with a cumulative incidence at 12 years of 14%. While this incidence is high, there were no SEER data available for direct comparison. These skin tumors seemed HCT-specific and were statistically significantly associated with a history of chronic skin GVHD, confirming previous reports [9, 10]. The connection between chronic GVHD and such skin tumors is not entirely clear but is likely the results of continuous repair of GHVD-related, immune-mediated skin damage as well as the long-term use of immunosuppressive drugs to treat chronic GVHD. Fortunately, if detected early, these skin tumors can be successfully removed by surgery.

Our analysis further showed that long-term survivors given non-myeloablative conditioning are at higher risk for solid SMNs other than non-melanoma skin cancers; 86 patients (5%) among the 1720 long-term survivors developed SMNs. However, this increased risk can almost exclusively be attributed to excess uterus (n = 2) and mucosal lip (n = 5) and penile (n = 2) carcinomas; the latter 2 tumors were predominantly seen among patients with chronic GVHD. All other SMNs had incidences which were not statistically significantly different from those seen in the general population.

How do our data compare to those reported previously? Most previous studies focused on patients given myeloablative conditioning regimens and, perhaps not surprisingly, reported statistically significant increases in the incidence of SMNs among HCT recipients. An analysis conducted by Baker et al. included patients receiving 2 to 4.5 Gy TBI [11] and found an incidence of SMNs comparable to that in patients given myeloablative chemotherapy alone, although still two-fold higher than in the general population. A cohort of patients was shared between Baker et al. analysis and our current study. While the analysis conducted by Baker et al encompassed patients who were transplanted only until 2014, our study includes patients up to 8/2023. It’s important to highlight that the upper limit for TBI doses in the study by Baker et al. was set at 4.5 Gy, while the TBI dose in our study ranged from 2–3 Gy. As the previous study [11] indicated, an increasing dose of TBI increased the risk of SMNs significantly.

Basal and squamous cell skin cancers were the most frequent tumors with a 1.5 times higher hazard among patients with chronic GVHD (95% CI; [1.07, 1.99]). in our analysis. Unfortunately, a comparison with such cancers in the general population was not possible since they have not been tracked by cancer statistics. Immunosuppressed patients are at risk of developing cutaneous neoplasm [12] and immunosuppression is a mainstay of GvHD treatment. A meta-analysis including 50,951 HCT patients showed that chronic GvHD was associated with increased incidences of squamous-cell and basal-cell carcinomas but had no demonstrable effect on melanoma, and acute GvHD alone was not definitively associated with increased risk of skin cancer [13]. Another large case-controlled study of 24,011 HCT patients also reported that the risk of squamous-cell carcinoma was almost three-fold higher in patients with chronic GVHD [10] as did several other studies [14, 15].

As for the remaining tumors, we observed 93 tumors among our patients compared to an expected 73.4 tumors among the general population with a standard incidence ratio of 1.3. This statistically significant difference was solely due to two types of tumors, non-cutaneous squamous cell carcinomas (specifically lip carcinomas (5 cases) and penile carcinomas (2 cases)) and uterine carcinoma (2 cases).

Finding increases in lip and penile cancers was not unexpected, as these tumors have been linked to chronic GVHD in a previous study among patients with aplastic anemia undergoing allogeneic HCT [9]. Also, earlier research has suggested that chronic GVHD was a significant contributing factor to the development of non-cutaneous squamous/basal cell cancers [11, 16]. Oropharyngeal malignancies commonly arise in regions that were previously impacted by chronic GVHD, or in rare situations, simultaneously affected by it [10, 17]. 4 out of 5 patients with lip cancers and 1 of the 2 cases of penile cancers in our study had a history of chronic GVHD. The case with penile cancer who did not have chronic GVHD had history of genital human papilloma virus infection (unknown types).

There were 2 cases with uterus cancers reported in our cohort. The small number of observed cases could a spurious finding. Uterine cancer is primarily a disease of postmenopausal women, and consequently age is the most important risk factor [18]. The two current cases occurred at 2- and 9-years, respectively, after HCT. Both patients were 70 years old. The two patient who developed uterine cancer at 2 years had a history of both breast and thyroid cancers before -HCT. Tamoxifen treatment for her breast cancer might have also been a risk factor for uterine cancer [19]. The second patient had surgical treatment and has remained in long-term remission.

Our analysis indicates that although these long-term survivors face a greater likelihood of SMNs in comparison to the general population (p = 0.03), the nonmyeloablative HCT procedure is relatively safe, particularly since two of the three tumor types that demonstrated an increase—lip and penile (non-cutaneous basal/squamous cell) cancers—are associated with chronic GVHD and its treatment, thereby linking them to the allograft and the conditioning regimen; nonetheless, these tumors are also highly treatable. It is essential for physicians to consistently promote SMN screening for HCT survivors, with particular emphasis on skin and mucosal squamous-cell and basal-cell carcinoma. Further research incorporating systematic data collection and thorough reporting, along with extended follow-up, is required to accurately define the incidence and actual risk of developing skin cancer (and other SMNs) within this patient population.

## Figures and Tables

**Figure 2:**
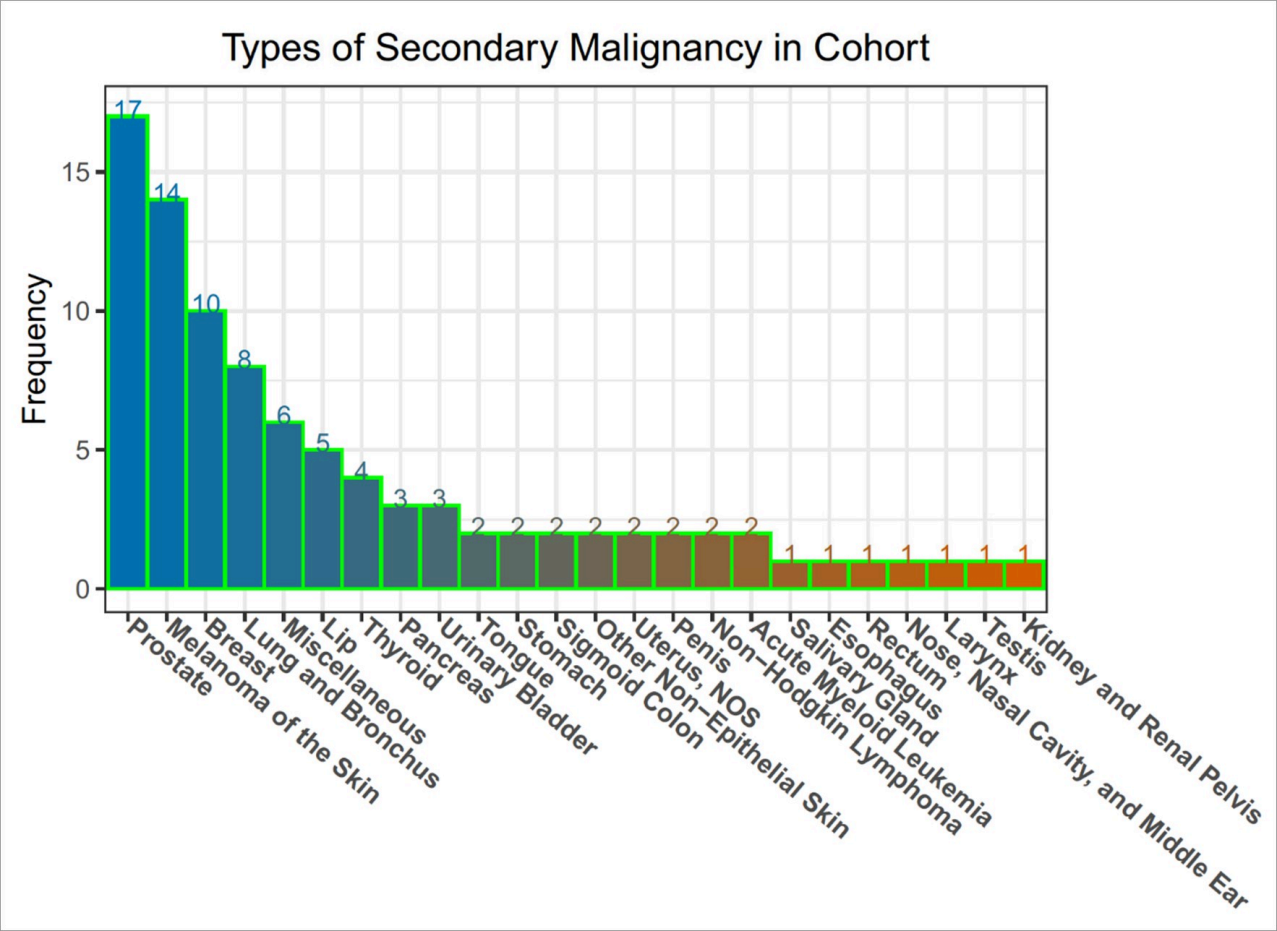
Types of SMNs

**Table 1:** Patient characteristics (N = 1720)

Characteristics	No (%)
**Median Age in years (range) at transplant**	59.7 (2.6–80.9)
**Sex**	
Male	1033 (60.1%)
Female	687 (39.9%)
**Conditioning Regimen**	
FLU, TBI (2 or 3 Gy)	1264 (73.5)
Rituximab, FLU, TBI (2 or 3 Gy)	36 (2.1)
CY, FLU, TBI (2 or 3 Gy)	213 (12.4)
Clofarabine, TBI (2 or 3 Gy)	53 (3.1)
TBI (2 or 3 Gy)	154 (8.9)
**Diagnosis at transplant**	
Hodgkin disease (HD)	109 (6.3%)
Non-Hodgkin lymphoma (non-HD)	308 (17.9%)
Acute Myeloid Leukemia (AML)	570 (33.1%)
Myelodysplastic syndrome (MDS)	270 (15.7%)
Multiple Myeloma (MM)	214 (12.4%)
Acute lymphoblastic leukemia (ALL)	112 (6.5%)
Chronic Lymphocytic leukemia (CLL)	137 (8.0%)
**Donor Type**	
Unrelated	1039 (60.4%)
Related	681 (39.6%)
**Donor/Graft Source**	
PBSC	1560 (90.7%)
Marrow	104 (6.0%)
Cord Blood	56 (3.3%)
**Match**	
HLA-Matched	1496(87)
HLA-Mismatched	224 (13)
**GVHD prophylaxis**	
CNI + SIRO + MMF	263 (15.3)
CNI + MMF	1323 (76.9)
CY + CNI+/-MMF	94 (5.5)
CY+CNI + SIRO	40 (2.3)

FLU, Fludarabine; TBI, Total body Irradiation; CY, Cyclophosphamide; CNI, Calcineurin inhibitors; SIRO, Sirolimus; MMF, Mycophenolate Mofetil.

**Table 2:** The observed number of SMNs compared to the Standardized Incidence Ratio (SIR).

Malignancy Class	Observed	Expected	SIR	P-value	95% CI
Acute Myeloid Leukemia	2	0.60	3.2	0.258	[0.39, 11.63]
Breast	10	16.80	0.6	0.107	[0.29, 1.09]
Esophagus	1	0.90	1.2	> 0.99	[0.03, 6.54]
Kidney and Renal Pelvis	1	2.80	0.4	0.466	[0.01,2]
Larynx	1	0.50	1.9	0.813	[0.05, 10.68]
Lip	5	0.20	25.3	< 0.001	[8.21, 59.01]
Lung and Bronchus	8	10.30	0.8	0.601	[0.34, 1.53]
Melanoma of the Skin	14	9.30	1.5	0.181	[0.82, 2.52]
Miscellaneous	6	2.60	2.3	0.102	[0.84, 4.98]
Non-Hodgkin Lymphoma	2	3.60	0.6	0.597	[0.07, 1.99]
Nose, Nasal Cavity, and Middle Ear	1	0.10	8.3	0.227	[0.21, 46.32]
Other Non-Epithelial Skin	2	0.40	4.6	0.144	[0.55, 16.51]
Pancreas	3	2.10	1.4	0.694	[0.3, 4.2]
Penis	2	0.10	15.1	0.016	[1.82, 54.37]
Prostate	17	11.90	1.4	0.187	[0.84, 2.3]
Rectum	1	1.60	0.6	> 0.99	[0.02, 3.45]
Salivary Gland	1	0.20	5.4	0.338	[0.14, 30.09]
Sigmoid Colon	2	1.30	1.5	0.751	[0.19, 5.53]
Stomach	2	1.10	1.9	0.574	[0.23, 6.8]
Testis	1	0.40	2.4	0.672	[0.06, 13.6]
Thyroid	4	2.10	1.9	0.311	[0.53, 4.95]
Tongue	2	0.80	2.6	0.359	[0.32, 9.42]
Urinary Bladder	3	3.70	0.8	0.993	[0.17, 2.38]
Uterus, NOS	2	0.02	87.7	0.001	[10.62, 316.87]
Total	93	73.40	1.3	0.031	[1.02, 1.55]

**Table 3:** Patients with more than 1 SMNs (excluding basal cell and squamous cell skin cancers)

Patient	Site and time (days post-HCT) of SMNs
1	Prostate (680 days) and Colon (3219 days)
2	Diffused large B cell Lymphoma (971 days) and MDS (1295 days)
3	Uterus (775 days) and Thyroid (1051 days)
4	Melanoma (127 days) and Pancreas (2126 days)
5	Lip (908 days) and Rectum (2443 days)
6	Urinary bladder (1534 days) and Lung (1898 days)
7	Melanoma (3961 days), and Pancreas (4681 days)

## Data Availability

For original, de-identified data, please contact the corresponding author (ptvo@fredhutch.org).
